# Commentary: Implantable hemodynamic monitoring: A potential milestone in left ventricular assist device management

**DOI:** 10.1016/j.xjon.2021.10.012

**Published:** 2021-10-22

**Authors:** Francesco Formica

**Affiliations:** Department of Medicine and Surgery, University of Parma, Parma, Italy; Cardiac Surgery Clinic, University Hospital of Parma, Parma, Italy

**Figure:**
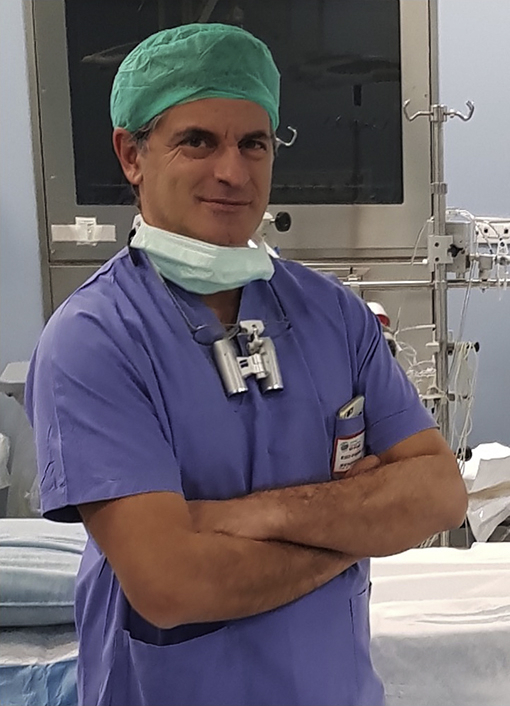
Francesco Formica, MD

**Central Message**

Implantable hemodynamic monitoring may be considered a milestone in the management of patients supported by LVAD because monitoring may dramatically improve the prognosis of these patients.

See Article page 18.

Heart failure is an advanced disease with an increasing influence on patient quality life, and the health system. These patients become increasingly frail and hemodynamically more unstable.

These factors are major causes of repeat hospitalizations and influence a patient's life by being associated with poor prognosis, poor quality of life, and reduced life expectancy. In advanced and extreme conditions (Intermacs stage 2 or 3), these patients no longer respond to medical therapy and are often kept hospitalized to undergo intensive medical treatment with intravenous inotropic drugs while waiting to undergo heart transplantation or left ventricular assist device (LVAD) implant. During the past decade, more and more attention has been paid to the use of continuous remote implantable hemodynamic monitor (IHM) to prevent both rapid deterioration of the hemodynamic parameters of patients with heart failure (HF) and to monitor continuously the course of patients assisted with LVADs. There is an increasing interest in the use of a wireless IHM (CardioMEMS; Abbott Labs, Abbott Park, Ill) in these patients because the CardioMEMS Heart Sensor Allows Monitoring of Pressure to Improve Outcomes in NYHA Class III Heart Failure Patients randomized trial demonstrated an advantage of this device in reducing significantly the rate of readmission compared with patients without a device (hazard ratio, 0.63; 95% CI, 0.52-0.77).^1^

CardioMEMS is positioned in 1 pulmonary artery branch during right heart catheterization and measures the pulmonary artery pressure and the filling pressures. It works according to the hypothesis that filling pressures will increase before other signs of decompensated HF occur. In addition, the usefulness of CardioMEMS is also likely to be found in patients with an LVAD. A decrease in filling pressures recorded by the device is frequent in situations such as hypovolemia and hemorrhage, whereas an increase in filling pressures is often associated with increased pulmonary congestion (as in case of LVAD thrombosis or aortic valve insufficiency) and in case of cardiac tamponade. Moreover, patients with fixed pulmonary hypertension before LVAD implant can be carefully monitored to verify the trend of pulmonary pressure during LVAD therapy to consider the patient eligible for heart transplant.^2^^,^^3^

Lampert and Teuteberg^4^ have presented a timely literature review regarding the use of CardioMEMS and the related results. The authors correctly emphasize IHM as a more accurate tool than echocardiographic evaluation in optimizing LVAD speed and in titrating diuretics and vasodilators. Based on the review and considering the relevant clinical utility of CardioMEMS, use of the device should be encouraged in many HF centers. We should recognize some limitations of the CardioMEMS. The high cost limits its wider use in clinical practice. Moreover, the device can measure only the pulmonary pressure, which maybe suboptimal in some scenarios such as primary pulmonary disease or increased pulmonary vascular resistance. In those scenarios, hemodynamic parameters such as pulmonary capillary wedge pressure of left atrial pressure can assess more appropriately the hemodynamic status and provide LVAD optimization.

CardioMEMS represents a promising tool and should be considered a cornerstone in the continuous monitoring of patients with HF and those assisted with an LVAD, especially because it could improve their prognosis.
